# Bridging the divide in digital therapeutics (DTx): Partnership strategies for broader representation across DTx development and deployment

**DOI:** 10.1371/journal.pdig.0001241

**Published:** 2026-02-20

**Authors:** Meelim Kim, Steven De La Torre, Uchechi Mitchell, Blanca Melendrez, Heather Cole-Lewis, Dana Lewis, Antwi Akom, Tessa Cruz, Bonnie Spring, Eric Hekler

**Affiliations:** 1 Herbert Wertheim School of Public Health and Human Longevity Science, University of California, San Diego, La Jolla, California, United States of America; 2 Center for Wireless & Population Health Systems, Calit2’s Qualcomm Institute, University of California, San Diego, La Jolla, California, United States of America; 3 The Design Lab, University of California, San Diego, La Jolla, California, United States of America; 4 School of Information, University of Michigan, Ann Arbor, Michigan, United States of America; 5 Division of Community Health Sciences, School of Public Health, University of Illinois Chicago, Chicago, Illinois, United States of America; 6 Center for Community Health, Altman Clinical and Translational Research Institute, University of California, San Diego, La Jolla, California, United States of America; 7 Google, Mountain View, California, United States of America; 8 OpenAPS, Seattle, Washington, United States of America; 9 The Social Innovation and Universal Opportunity Lab, Streetwyze, University of California San Francisco & San Francisco State University, San Francisco, California, United States of America; 10 Department of Preventive Medicine, Feinberg School of Medicine, Northwestern University, Chicago, Illinois, United States of America; Boston Consulting Group, KENYA

## Abstract

While Digital Therapeutics (DTx) are widely considered a key strategy to reach certain populations with unmet healthcare needs, a range of differences in the impact and adoption of DTx still exists. These differences are not just rooted in access, but also in gaps in knowledge about how to produce community-relevant DTx, primarily stemming from the implicit or explicit exclusion of those with both relevant trained expertise (gained through formal education or professional experience) and lived expertise (gained through personal and direct experience). This paper expands the traditional conceptualization of the digital divide beyond access to encompass four interconnected domains: the Digital Knowledge Divide, Digital Evidence Generation Divide, Digital Production Divide, and Digital Adoption Divide. Drawing on Ridgeway’s cultural schema theory of status, we demonstrate how conventional team hierarchies in DTx development systematically allocate status and decision-making authority through automatic cultural defaults, credentials, professional roles, demographic characteristics, rather than through contextual assessment of who possesses the most relevant expertise for specific decisions. To address this challenge, we propose a theoretical framework for dynamic expertise integration that deliberately disrupts rapid-stabilizing hierarchies by creating explicit relational spaces where teams can recognize and value both lived and trained expertise contextually. We operationalize this framework through the DTx Team Building Worksheet, a practical tool that integrates team science approaches with Community-Led Transformation principles and Culturally and Community Responsive Design. The Worksheet provides structured processes for assessing diverse forms of expertise, defining roles dynamically, and identifying decision-making priorities that shift appropriately across the DTx lifecycle. This integrated approach including problem analysis, theoretical framework, and practical tool, offers a pathway toward more equitable DTx development by enabling teams to make status dynamics explicit, expand what counts as expertise, and establish new consensual norms about contextually-appropriate status allocation. We invite stakeholders across sectors to test and refine these tools in diverse contexts, recognizing that creating equitable DTx requires sustained commitment to partnerships that genuinely honor multiple forms of expertise and willingness to disrupt comfortable hierarchies in service of producing interventions truly designed for and with the communities they aim to serve.

## Author summary

In recent years, digital health tools like Digital Therapeutics (DTx) have gained attention as a promising way to support health behaviors across different communities. However, there are still major gaps in how well these tools serve diverse populations. The problem isn’t just about who has access to technology, it’s about who gets to shape how these tools are designed and whose knowledge is valued in the development process. DTx development teams often automatically give more decision-making power to people with formal credentials while overlooking the essential expertise that comes from actually living with a health condition or navigating real-world barriers to care. We offer a practical strategy to address this gap: a worksheet-based method that helps teams recognize and value both lived expertise (knowledge from personal experience) and trained expertise (knowledge from formal education). The key insight is that the most relevant type of expertise changes depending on what decision is being made. By making these decisions more transparent and dynamic, teams can create digital health tools that actually work for the people who need them most. We’ve seen this approach succeed in our own collaborations outside of DTx, and we’re inviting others to try it and help build a future where digital health tools genuinely serve everyone.

## Background

### The promise of digital therapeutics (DTx)

One of the key hopes and promises of digital health tools is the possibility that they could be used to help provide quality care to those who may struggle to receive care from traditional healthcare systems. This is embedded in the logic of Digital Therapeutics (DTx), which are defined as software-based health tools engineered to prevent, treat, or manage diseases, disorders, conditions, or injuries. These tools are characterized by their ability to deliver therapeutic interventions that have proven beneficial effects on individual health and result in measurable real-world outcomes [1,2]. While there is great promise in DTx, to date, the reality of the situation is quite different, with a range of differences in the production and adoption of community-relevant DTx [3]. This gap is often described in terms of digital divides, a concept traditionally used to refer to lack of access to the internet, digital tools, or devices such as phones and computers [4]. In our context however, the notion of digital divides can be understood more broadly, a central point we will elaborate on further in this work. While there are many possible ways to reduce these differences, we propose that robust, dynamic, mutually beneficial partnerships between those with trained expertise (i.e., gained through formal education or professional experience such as someone with a degree in computer science) in developing and implementing effective tools and those with relevant lived expertise (i.e., those who have gained personal and direct experience in facing challenges accessing healthcare and maintaining good health) are needed to have all of the relevant knowledge, skills, and wisdom involved in a team to allow it to create solutions that can work in real-world contexts.

### Purpose

This paper grew out of our shared commitment to advancing community partnerships in the development of digital therapeutics. Our author team brings together individuals with diverse lived and trained experiences from community, academic research, and technological industry settings. These varied forms of expertise shaped how we conceptualized community engagement, identified barriers to inclusion, and developed the framework and tools offered in this work. We acknowledge that our own professional and lived perspectives influence the lens through which we approach inclusion and community partnerships in digital health, and we aimed to make those commitments explicit throughout this work. With this orientation in mind, this paper proposes a set of practical tools to guide community partnership formation and evolution, informed by prior work, existing theory, and our real-world experiences of building partnerships.

We first summarize the literature regarding the current state of digital divides in DTx design, production, and adoption, to better define the problem we aim to address. Next, we discuss why developing robust processes to foster effective partnerships is a viable hypothesis for how we might create and refine tools focused on improving partnerships processes to address these digital divides. To this end, we discuss how we incorporated community-centered design principles and practices. First, we drew on Community-Led Transformation (CLT) principles [5], a suite of nine practice principles co-developed over more than 20 years by the University of California San Diego Center for Community Health (CCH) in collaboration with community-based and ethnic-led partners, including the San Diego Refugee Communities Coalition. These published principles reimagine learning and public health systems by authentically centering community-based organizations as equal and essential partners in design, leadership, and decision-making. We also incorporated Culturally and Community Responsive Design (CCRD) [6], which centers individual and community assets, behaviors, and needs throughout different stages of the design process, as well as Team Science approaches [7,8], which leverages diverse expertise to address complex problems. We then summarize the limitations of this work, most critically, that these tools, although justified by the references mentioned earlier, have not yet been rigorously vetted. We conclude with suggested next steps, specifically an invitation for members of the community, researchers, and designers to actively try out, critique, challenge, and ultimately refine these tools.

## Part 1. The digital divides

### The current state of digital divides in DTx: Access and beyond

Access to traditional healthcare and related resources is informed by historical and systemic influences including factors at the structural, environmental, and societal levels, which ultimately impacts individuals and communities differently. These factors create conditions where some communities have greater access to healthcare services, while others experience more obstacles, ultimately leading to differences in long term health outcomes [9,10]. These factors, often referred to as Social Determinants of Health (SDoH), include factors such as access to quality education, experiences of economic resource scarcity (including experiences of unhousing and food insecurity), and living, working, and growing in environments that make it difficult for certain individuals and communities to have opportunities to access healthcare and maintain good health [11–13]. In the context of DTx, there is a documented digital divide that extends these traditional issues communities face with accessing healthcare, into the space of access to digital health care technology (including DTx). However, the digital divide itself as it was originally specified in terms of access, has expanded further into several upstream domains, primarily manifesting in a divide in DTx knowledge and production, which we suggest is primarily stemming from the lack of inclusion of community members with relevant lived expertise in the DTx design and production phases, ultimately leading to poor person-level uptake and engagement [14–17].

As previously mentioned, the digital divide was initially defined in relation to differential access to mobile technology and the internet, particularly in the form of home broadband. While still present for some groups such as those who live in rural settings [18] and those with lower household income [19], the traditional digital divide in terms of access has improved for many other groups. For example, members of racial/ethnic minority groups in the U.S. own smartphones in comparable rates to their non-Hispanic White counterparts (91% ownership), particularly among Hispanic/Latinos (91%), and African Americans (84%) [20,21]. In fact, smartphone ownership is high even amongst communities often referred to as “difficult-to-reach” subpopulations such as migrant farmworkers of Hispanic/Latino background [22], those with reported lower household-income living in urban settings which are densely-populated [23], and those classified by healthcare agencies as “safety net” populations [24]. Furthermore, members of racial/ethnic minority groups in the U.S. are more likely to be “mobile only” internet users which means they primarily access the internet from their personal smartphone, which indicates a shift in focus of internet access away from home broadband [20,25]. These shifts increase the possibility for DTx to become a viable option for communities facing challenges with access to healthcare and maintaining good health. However, there remains digital divides evolving from the implicit or explicit exclusion of community members in DTx research and development which, we suggest, has led to the lack of production of digital tools that are appropriate for sub-groups explicitly experiencing health and healthcare challenges.

Our exploration of the digital divide in digital therapeutics (DTx) aligns with recent research showing that this divide goes beyond basic access issues to include differences in digital literacy and the use of algorithms and AI [26]. These findings are echoed in other studies as well that find that these differences in DTx usage are rooted in both digital literacy and, as we contend, a person’s socio-economic context, which are central to understanding digital divides [27]. Ultimately, this work shows that digital therapeutics can worsen health disparities due to gaps in usage and knowledge, making DTx a potential barrier to a good healthcare system rather than a bridge, despite good intentions.

**Fig 1:**
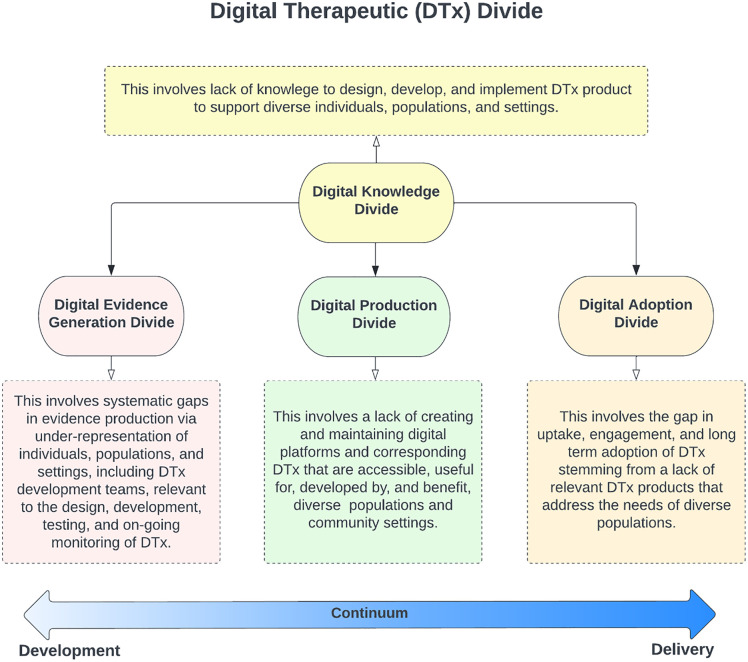
The definitions of expanded digital divides.

In this paper, we describe the current state of DTx digital divides which we distill into four areas, 1) Digital Knowledge Divide; 2) Digital Evidence Generation Divide; 3) Digital Production Divide; and 4) Digital Adoption Divide (see Fig 1).

#### Digital knowledge divide.

This refers to a gap in the available knowledge across the continuum from development to delivery of community relevant DTx products. We describe this divide as stemming from three other divides, as follows.

#### Digital evidence generation divide.

By digital evidence generation divide, we refer to historic differences in the degree to which people who experiencing challenges accessing healthcare and maintaining good health are actively involved in, guide, and, when appropriate, lead DTx scientific pursuits towards evidence generation that could be used to directly benefit people like them. This exclusion happens despite their receptivity to participating in digital health research [28,29] resulting in a clear lack of inclusion [30]. This failure of scientific studies to convert participant willingness into participation often stems from barriers including narrow recruitment channels, such as studies recruiting from primarily academic and clinical settings (instead of, for example, recruitment occurring within and by community members themselves for one another) as well as impacts of eligibility criteria (e.g., excluding persons who are not English proficient). A systematic review that looked at 83 manuscripts aiming to characterize the design and impact of mobile health applications (mHealth) among individuals from low socio-economic backgrounds and racial/ethnic minority groups found that the diversity among people in these studies who can benefit from DTx was not reflected in the samples, including a gap in the inclusion of people from Native American and First Nation populations, underrepresentation of Males, non-English speaking populations, and those who live in rural settings [31].

In the context of algorithms, machine learning, and AI (components often included in DTx), differences in data representation and algorithmic awareness are prevalent. When digital health data used for developing and validating DTx predominately represents the behaviors and health contexts of more digitally literate or more economically resourced populations. Such omissions can prevent the foundational evidence base from reflecting real-world community knowledge, wisdom, and needs [27]. This divide limits the relevance and effectiveness of DTx for underrepresented communities, ultimately skewing clinical outcomes and propagating differential health outcomes. Ultimately, we posit that the current lack of inclusion of community members in DTx evidence generation results in a lack of relevant DTx products that reflect the unique knowledge, needs, and desires of these populations.

#### Digital production divide.

Although previous research has highlighted the importance of a user-centered approach to DTx design and development, current commercially available DTx products do not meet the language, digital literacy, cultural relevance, and broader social needs of real-world communities facing healthcare challenges and maintaining good health, arguably those who might benefit the most from their usage [32–35]. Furthermore, research has shown that taking DTx products that are exclusively in the English language and translating the material into a user’s native language (i.e., Spanish) has not been found to positively influence the uptake and engagement of mHealth applications, citing the need to move beyond solely translation efforts [35]. Based on this, it is unlikely that current approaches, such as professionally driven, user-centered design, are sufficient to guide the production of DTx to be appropriate for various populations, thus limiting the likelihood of real-world impact and adoption. Consequently, if those developing DTx do not include evidence from those who will be using them in real-world settings, products may inherently be better suited for those with higher tech literacy, leaving community members with different levels of tech literacy behind. Furthermore, algorithm-driven personalization within DTx can unintentionally reinforce these gaps if training data lacks relevance to reflect the community the DTx is designed for, highlighting the need for the inclusion of those with community relevant knowledge and expertise.

#### Digital adoption divide.

Evidence suggests that people with more resources have more opportunities to encounter, attend to, and retain information about DTx than those with less resources, particularly among those who experience challenges accessing traditional healthcare. The literature underscores that access alone is insufficient, while differences in necessary literacy, trust, and consistent usage directly impact who can meaningfully benefit from DTx [26,36]. Due to these factors, currently available DTx have low user uptake and engagement among communities who have historically had difficulty accessing and using traditional healthcare. For example, research has found that Hispanic/Latinos are 0.63 times less likely to download mHealth applications compared to their non-Hispanic/Latino White counterparts (OR 0.37 95% CI 0.20-0.69 p = .002) (n = 848) [37]. Another study of a fully remote clinical trial utilizing mHealth applications found that Hispanic/Latinos were more likely to drop out two weeks earlier than their non-Hispanic/Latino counterparts (n = 345). In this study, Non-Hispanic/Latino individuals tended to participate in the study for 18.5 days longer than their Hispanic/Latino counterparts (median 53.5 days until dropout for non-Hispanic/Latinos compared to a median of 37 days for Hispanic/Latino participants) [38]. Ultimately, to work towards increasing digital adoption and uptake among different communities, there is a need to address divides in both evidence production and product development, as each reinforces the other: limited evidence constrains product development, and limited products further constrain evidence generation. Our work supports the view that without addressing these systemic and person-level usage barriers, DTx may exacerbate, rather than alleviate, existing health and healthcare challenges and differential health outcomes.

### Real world impact of the current digital divides

There are significant health and healthcare implications for the populations impacted by these digital divides in DTx. For example, cardiovascular disease (CVD) remains the leading cause of death among most Americans and is higher among racial/ethnic minority groups [39]. There is also a documented lack of access to CVD care and information, particularly among African American populations [40]. The use of DTx has shown potential for improving CVD health outcomes among these populations [40]. However, given the digital divides discussed above, there is a missed opportunity to positively impact CVD among these racial/ethnic minority groups. The same argument applies to other health conditions including dermatological care [41], mental health [42], and chronic diseases including diabetes and obesity [43]. Taken together, the current state of DTx digital divides weakens the current promise of DTx to reduce healthcare challenges. While there are many possible strategies for addressing this, we suggest that DTx developers consider more dynamic, robust, and transparent partnerships with individuals and communities that possess the relevant lived and/or trained expertise to design, develop, and implement relevant DTX that can meet their real-world needs. In the next section, we will justify this proposed emphasis on robust partnerships.

## Part 2. A status-based framework for dynamic expertise integration

The development of equitable digital therapeutics requires groups built on robust partnerships that can effectively integrate multiple forms of expertise. However, traditional hierarchies in health technology development systematically privilege certain types of knowledge, particularly credentialed clinical and technical expertise, while devaluing or marginalizing others, especially the lived expertise of patients and community members. Drawing on Ridgeway’s cultural schema theory of status [44], we argue this pattern represents not merely an ethical failing but a fundamental inefficiency in goal attainment that undermines the quality of DTx interventions.

Ridgeway defines status as “a comparative social ranking of people, groups, or objects in terms of the social esteem, honor, and respect accorded to them”. She argues that status hierarchies emerge ubiquitously because they address a fundamental tension in cooperative endeavors: people are interdependent in achieving shared goals yet also have competing interests in maximizing personal benefits and influence. In goal-oriented groups, status hierarchies form within minutes and stabilize quickly, allocating esteem and authority to those perceived as offering greatest value to the shared effort.

Critically, Ridgeway demonstrates that status is fundamentally given, not taken; it depends on how others within a group evaluate and respond to each member. This creates a multilevel relational dynamic: individual actors (first-person perspective) interact within specific relationships (second-person dynamics) while simultaneously being influenced by shared cultural beliefs (third-person norms) about what and who “counts” as valuable. These third-person norms/cultural status beliefs function as “common knowledge” that allows group members to coordinate their judgments about who deserves esteem and influence. At the same time, they bring broader societal hierarchies based on credentials, professional roles, race, gender, and socioeconomic status into team dynamics.

In typical DTx development contexts, these third-person cultural factors automatically confer higher status on clinicians, researchers, and technology developers while systematically devaluing the contributions of patients and community members. This occurs even when the shared goal of developing effective, equitable interventions would be better served by honoring multiple forms of expertise. Ridgeway would describe this as a misalignment between status allocation and actual contribution to collective goals. Teams often privilege credentials over contextual knowledge, leading to interventions that fail to address real-world implementation barriers, overlook accessibility constraints, and ignore the experiential wisdom of those navigating health systems and conditions. As a result, DTx development processes risk reproducing rather than reducing health inequities.

### Expanding what counts: Recognizing lived and trained expertise as distributed assets

Building on this status-based framework, we begin from a foundational recognition that every team member brings potentially relevant expertise derived from both their lived experiences and their training. A community member might bring not only experiential knowledge of navigating Type 1 diabetes but also programming expertise from their professional work. A researcher might bring both methodological training and lived experience growing up in an underserved community. A patient might bring both experiential knowledge of their condition and professional expertise in user experience design. Traditional status hierarchies that rely on singular identity categories such as “the researcher,” “the community member,” or “the clinician” obscure this multidimensional reality.

**Fig 2:**
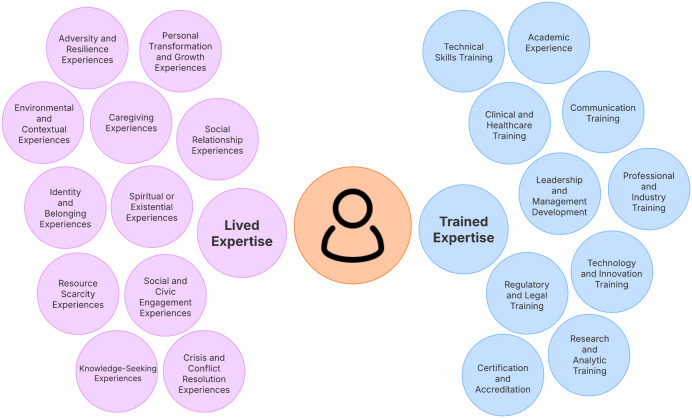
Examples of lived and trained expertise.

Trained expertise includes specialized training and knowledge, which is typically gained through study, training, or practice [45]. This form of experience often leads to educational degrees or work experience in a specific scientific field resulting in the person’s inclusion in research efforts as “subject matter experts” in those areas. On the other hand, lived expertise refers to personal knowledge rooted in direct experience. It functions as a type of “tacit knowledge”; the type of experiential knowledge that is not easily reducible to concept, terms, or other representations [46]. Ultimately, lived expertise brings a unique and holistic perspective shaped by the totality of a person’s experiences, not solely by membership in a specific sociodemographic group. It reflects the accumulated understanding gained from navigating real contexts over time (see Fig 2).

All persons have varying degrees of lived and trained expertise relevant to a given DTx goal or purpose. For example, it is quite possible for someone to both have a doctoral degree in behavioral science with advanced knowledge of the DTx design, development, testing, and monitoring, while also being of Mexican-American origin who grew up in Boyle Heights. Such a person combines technical expertise with lived expertise and experiential knowledge of a historically underserved community of Los Angeles. We highlight this to emphasize that lived and trained expertise are likely best thought of as distinct facets of relevant knowledge, skills, and practices that a person can leverage in the design, development, and implementation of DTx. However, these forms of expertise, particularly those rooted in experiential and relational ways of knowing, are under-recognized and under-utilized in relation to DTx production. To illustrate this, see Fig 2, which is meant to highlight various dimensions of possible lived and trained expertise domains that every person on a team will have varying levels of expertise in.

Returning to the issue of digital divides, we argue that equitable DTx development requires a far broader range of expertise than is typically acknowledged. For instance, developing a diabetes management DTx depends not only clinical and technical knowledge of software development, but also experiential knowledge of living with the condition. It further requires understanding of health insurance billing codes and reimbursement structures; awareness of accessibility needs for users with varying literacy levels or visual impairments; knowledge of cultural factors that shape health practices in different communities; understanding of the structural barriers (e.g., unstable housing, food insecurity) that shape technology adoption; and wisdom about when introducing technology might inadvertently marginalize rather than help specific populations.

No single individual possesses all these forms of knowledge. Rather, they are distributed across team members with different backgrounds, each bringing expertise shaped by both training and lived experience. The challenge, then, is creating team processes that can recognize, value, and effectively integrate this distributed expertise. Doing so requires deliberately disrupting the rapid stabilization of conventional status hierarchies.

### The limitations of current partnership/team formation & management approaches

The importance of robust, multisector partnerships in DTx development has been identified in previous literature and presents an opportunity to bring together those from community, academia, and industry [47–49]. While these entities may approach DTx development differently, the strengths and benefits of each perspective can be leveraged. For example, when working from a more classical academic approach, there is an emphasis on applying behavioral theory and existing research to design and develop DTx for specific populations with specific needs. When using approaches used in industry, there is a focus on developing DTx products for broad audiences that can be refined through user experience testing and feedback [50]. When community groups engage in this line of work, there is a focus on describing and elevating the needs of the community to ensure success criteria are relevant and will have real-world impact for the people who are supposed to benefit from the DTx.

Although these sectors approach DTx development differently, most research on group or partnership composition focuses primarily on using third-person social norm concepts, such as demographic characteristics (e.g., race/ethnicity, gender, socioeconomic status; [51,52]). Additionally, typical group compositions, including those for scientific investigation and technology development, predominantly utilize a “staffing” approach [53]. In this framework, individuals are included in the group or partnership based on having a set of prerequisite requirements pertinent to the tasks, goals, or objectives of the group. Typically, this process prioritizes previous professional training experiences such as work history and educational attainment.

In these types of partnerships or groups there is typically an imposed hierarchical structure that fixes people into leadership or decision-making roles privileging those with higher trained expertise and backgrounds as opposed to those with lived expertise. An overarching assumption of this approach holds that people can be reduced to a singular identity (based on one set of demographic characteristics for example) and does not recognize the richness of an individual’s unique and dynamic lived experience and expertise. This approach also fixes the multiplicity of the group at the onset of a project and does not allow for dynamic or situational relevance to emerge when different expertise may be more relevant for different goals, tasks, or decisions.

From a status theory perspective, these conventional approaches allow third-person cultural norms about credentials and professional roles to automatically allocate status in ways that persist throughout a project, regardless of whether those with highest status possess the most relevant expertise for specific decisions. We hypothesize, based on our own experience and building on the work of Ridgeway, that this classical approach to team constructure, is a critical factor that might be leading to digital divides described above. If this is the case, there is a need to articulate alternative ways for guiding team formation and managing DTx development.

### Creating second-person space for dynamic status allocation

Ridgeway’s framework reveals why conventional status hierarchies are so persistent: they provide functional solutions to coordination problems, and once established (often within minutes), they create consensual expectations that both high- and low-status members recognize and typically reinforce through their behavior [44]. Disrupting these patterns requires more than good intentions; it requires creating explicit second-person relational spaces where teams can collectively examine and renegotiate what confers legitimate status in their specific context.

Our approach proposes making status dynamics explicit rather than implicit and establishing new consensual norms about what constitutes valuable contribution to the shared goal of developing equitable DTx. Honoring and strengthening the orientations of different sectors are critical for producing meaningful and impactful DTx. We further hypothesize that a healthy dialectic space that honors and fosters each person’s full array of relevant expertise, both lived and trained, can foster a team culture grounded in mutual respect and inclusion. Such a culture enables more dynamic allocation of status and, in turn, more dynamic types of decision-making relevant to DTx development. This involves several key shifts:

#### From automatic to contextual status allocation.

Rather than allowing cultural defaults (credentials, professional roles, demographic characteristics) to automatically confer status, we seek to foster teams that develop practices to determine which expertise is most relevant for each decision. This involves asking a central question: Who has the most holistic grasp of reality for this decision? The answer will differ across decisions, from high-level strategic choices to micro-level design details, and thus status and decision-making authority should flow dynamically rather than remaining fixed.

#### From singular to multidimensional recognition.

Instead of categorizing team members through singular identities that trigger conventional status hierarchies, we seek to foster teams that cultivate awareness of the multiple forms of potentially relevant expertise each person contributes. This includes creating opportunities for team members to articulate their diverse knowledge bases and for the team to develop shared understanding of when different types of expertise become salient. Thoughtful partnership processes can support this sectoral blending of perspectives by inviting individuals to share their relevant lived and trained expertise to a given issue. Together, teams can collaboratively co-design a DTx solutions that leverage the full range of expertise represented among members. At the same time, teams should remain mindful of additional types of expertise that may be needed but are not currently part of the team.

#### From implicit performance expectations to explicit expertise mapping.

Ridgeway demonstrates that people form often-unconscious “performance expectations” based on perceived status characteristics, which then become self-fulfilling as others defer to those expected to contribute more. We are seeking to cultivate teams that can interrupt negative performance expectations about those lacking conventional credentials while maintaining appropriate discernment about when technical or clinical expertise is genuinely necessary. This requires making explicit what expertise is needed for what decisions and who possesses relevant knowledge, rather than relying on automatic associations between credentials and competence.

#### From static hierarchy to dynamic influence.

While Ridgeway’s research shows that status hierarchies typically stabilize quickly and persist, in our various community work described above and at the end, we have found, in real-world practice, that it is possible to create second-person dialogical spaces that can enable this type of fluid pattern. Using this prior experience and this theoretical foundation as a guide, we have developed a draft protocol and structure to foster other teams in developing the capacity to support this dynamic status and, by extension, dynamic decision-making process. This includes recognizing that sometimes the appropriate role for formally credentialed team members is to listen, learn, and defer rather than to decide.

Building groups and partnerships based on this recognition of the combination of trained and lived expertise acknowledges an individual’s wide range of experiences and, we propose, can provide a pathway for collaborative co-designed DTx solutions that have the potential to be more relevant, acceptable, and effective compared to traditional DTx development approaches. Thus, we propose team formation processes as an under-examined intervention point that could be used to counteract the digital health divides outlined in the earlier section.

Valuing and centering lived experience flips the typical dynamic where knowledge and expertise on a problem traditionally comes from ‘professionals’, institutions, authorities, and governments that have studied or assessed a situation detached from direct experience. In a health context, persons with lived experience are regarded as ‘experts by experience’ in the scope of their first-hand experience with a diagnosis or health condition. Research suggests that including someone with relevant lived experience in a patient’s care and treatment has significant contributions to their experience of treatment and overall health outcomes [54]. Unfortunately, this is not a ubiquitous approach in clinical settings [54]. Creating partnerships that honor and nurture teams with diverse and relevant lived and trained expertise can provide a pathway for creating collaborative spaces for robust DTx co-design and development to occur that has the potential to address the new digital divides previously defined.

### The case for dynamic, expertise-based partnerships

The key reason for fostering robust, dynamic, authentic, and mutually beneficial partnerships between DTx developers and those with lived experiences is the need to have all the relevant knowledge, skills, and wisdom involved in a team to allow it to create solutions that can work in real-world contexts. Without such partnerships and robust dynamic teams, there is a high likelihood that decisions will be made based on incomplete knowledge or faulty assumptions. We contend that current strategies, such as using human-centered design approaches, are valuable, but insufficient, particularly when seeking to create systems that can work for those most in need. This aligns with universal design principles advocated by individuals with disabilities, suggesting that systems should ideally serve those facing the most acute healthcare challenges. DTx designed for the ‘normative person’ often fails to accommodate those facing the most acute challenges, making user-centered design critical, yet insufficient alone to prevent the digital divides that exclude many users [55].

What is needed instead is to integrate persons meaningfully and actively with lived expertise to be central members of the DTx development team, to ensure, throughout all stages of the process, persons with real-world knowledge are present when decisions are being made and actively deferred to and conferred authority and status when their lived expertise is most relevant to a given DTx development decision (described in greater detail below). We propose that addressing these issues requires robust, knowledgeable partnerships that utilize conscious and explicit 2^nd^-person team formation processes that can foster this type of dynamic status and decision-making. Critically, this work can be informed by prior work, including community-centered design principles and team science approaches, which have been reviewed to guide the development of the tools outlined in the last section of this paper [56]. Specifically, we contend that tools that aid in the identification of trained and lived expertise may enhance the development of multisector partnerships aimed at creating DTx products that have real-world impact in naturalistic settings (e.g., where people live and work).

### Success criteria: When status honors reality

How would we know if such a process is working? The ultimate measure is functional: does dynamic status allocation enhance the team’s ability to achieve its shared goal of developing equitable, effective DTx? More specifically, success would be evident when:

- **Decision quality improves** because relevant expertise, whether from training or lived experience, is consistently recognized and integrated. This stands in contrast to situations where certain voices are systematically privileged or marginalized regardless of their actual contribution to specific decisions.
- **Team members with lived expertise report** that their knowledge is actively sought, seriously considered, and visibly shapes decisions. At times, they may serve as the primary decision maker for issues most closely aligned with their experience, rather than being tokenistically acknowledged but functionally ignored.
- **Influence flows dynamically** across the team, guided by the question at hand, rather than concentrating persistently in those with conventional credentials or demographic privilege.
- **The team can articulate** which forms of expertise are relevant to which decisions and demonstrates awareness of how third-person cultural factors (stereotypes, assumptions about sectors, automatic deference to credentials) might be distorting their judgments.
- **Decisions reflect holistic understanding** that integrates clinical efficacy, technical feasibility, lived experience of target populations, implementation realities, accessibility constraints, and structural factors that shape technology adoption, rather than privileging one domain of knowledge over others.
- **The resulting DTx interventions** demonstrably serve diverse populations effectively, thus, showing progress on reducing the digital divides outlined in Part 1 of this paper.

Importantly, this is not a rejection of expertise, credentials, or specialized training. Technical knowledge matters; clinical expertise matters; methodological rigor matters. Rather, this framework expands what counts as expertise and insists that the relevance of any form of knowledge, whether from training or lived experience, must be assessed contextually in relation to specific decisions. The goal is a *dynamic meritocracy* where “merit” is understood as having the most robust sense of reality for a given decision, recognizing that reality is multifaceted and that comprehensive understanding requires integrating multiple ways of knowing.

### From theory to practice

This theoretical framework provides the foundation for the collaborative approach we describe in the last section. By recognizing how third-person cultural norms automatically shape status allocation in ways that undermine goal achievement, we can deliberately create second-person relational practices that enable teams to develop new consensual norms that honor the diversity of each individual and also help to highlight where forms of expertise, whether lived or trained, may not yet be sufficiently represented in the team/partnership. These norms honor the full range of expertise needed for equitable DTx development, acknowledging that the wisdom required to understand when, where, for whom, and under what circumstances a digital intervention will be genuinely helpful (rather than inadvertently marginalizing) is distributed across team members with diverse forms of both lived and trained expertise.

The challenge is substantial: we are asking teams to actively disrupt status hierarchies that Ridgeway shows typically stabilize within minutes and that serve real coordination functions. Yet our experience demonstrates this is achievable when teams commit to making status dynamics explicit, developing shared understanding of distributed expertise, and creating structures that enable influence and authority to flow toward whoever holds the most contextually relevant knowledge for each decision. The following sections describe how we have operationalized these principles in practice. We offer this as a prototype meant to combine both what we have gleaned from prior work, particularly Ridgeway’s work, and what we have learned in actively forming teams that have strong co-leadership structures that has community members as key co-leads.

## Part 3. Proposed contextualisation

This section outlines the processes described in the DTx Team Building Worksheet (hereafter referred to as ‘the Worksheet’), which is designed to support building robust partnerships and effective teams for the development and deployment of DTx. These processes can be applied at any stage of the Digital Therapeutics Real-World Evidence Framework (DTx RWE Framework) [57] to enhance its effectiveness and inclusivity. Additionally, it addresses the ongoing digital divide in DTx production, adoption, and sustained engagement. In the following section, we briefly introduce the DTx RWE Framework to provide an overview of the DTx lifecycle. We offer this to highlight the key set of “decisions” that need to be made across the lifecycle of a DTx. Subsequently, we detail the Worksheet (see S1 Text), our proposed tool for facilitating team development discussions from the outset of designing, developing, testing, and sustaining DTx that can manifest a team environment that dynamically confers status and, thus, decision-making authority, across the team, oriented around who, on the team, has the most holistic grasp of reality relevant for a given decision.

### Conceptual framework of DTx RWE

The DTx RWE Framework is a 4-phase model that utilizes a pragmatic, iterative, milestone-driven approach for DTx development to long-term use. The Framework incorporates real-world data (RWD) across the entire life cycle of DTx development and use to ensure the highest level of fidelity to the needs of users. To integrate RWD, the Framework points out honoring and fostering teams that include three key roles: DTx implementers; persons or groups who directly serve and know the communities being served; and those with methods expertise relevant to each phase. The Framework provides a structure and guidance on the types of activities, their roles and purpose, and the methods used for each. This paper complements this Framework by providing a set of processes and steps a group could use to facilitate the formation of teams for any phase of the Framework process. For example, in a funded project with Hispanic/Latino communities, we used these processes to convene a diverse team of community members to guide the co-design of a culturally responsive intervention. In the remainder of this, we will be drawing on terms and concepts from the Framework; for full definitions and articulation of these concepts, please refer to the DTx RWE Framework.

### Developing the worksheet based on prior work

Building on the DTx RWE Framework, our Worksheet is crafted by integrating the Team Science approach [8] with Community-Led Transformation (CLT) principles [5] and Culturally and Community Responsive Design (CCRD) [6], along with insights from real-world implementation of these practices across several projects led by our co-authors. The team science approach fosters collaboration across multiple specialties to address scientific questions, offering clear clinical and patient-centered advantages by incorporating diverse perspectives that aid in problem identification and resolution [7,8]. In alignment with this approach, our ultimate goal is to foster sustainable DTx solutions through robust partnerships that continue to evolve and flourish, supported by community assets and infrastructure.

By applying CLT principles, which emphasize deep community engagement, we enable communities to actively participate in decision-making, ensuring that decisions are made transparently and inclusively [5]. To be more specific, the Worksheet includes an initial decision-making protocol that prioritizes transparency and inclusivity by determining whose expertise is most relevant for each module at each stage. This structured approach aligns with CLT principles, ensuring that all decisions reflect a comprehensive understanding of community needs and strengths. By valuing both trained and lived expertise, the process supports a holistic approach to development, fostering respect and ownership among all team members.

Furthermore, the Worksheet incorporates the CCRD approach, prioritizing the cultural, social, and environmental specifics of community needs. This strategy ensures that the development of products is closely aligned with and supportive of community values, customs, and priorities [6]. For instance, during team formation, the Worksheet facilitates a deep dive into the specific needs and values of each team member and their associated groups, crafting a development pathway that respects and integrates diverse perspectives.

Alongside these design principles, insights from real-world projects further guided the development of the Worksheet. In the HEALthy 4 You (H4Y) project, for example, researchers from University of California San Diego (UCSD) partnered with Family Health Centers of San Diego (FHCSD), the San Diego County Childhood Obesity Initiative, Streetwyze, and eleven community-based organizations, to address adverse childhood experiences (ACEs) and childhood obesity among Latino families in San Diego [58]. The project has two key elements, one focused on advancing an eventual clinical trial in FHCSD and the other was explicitly focused on learning about community priorities to guide future public health practice efforts. To do the latter, the team invested in relationship-building and community story gathering via the Streetwyze platform, supported through parallel community engagement efforts within both H4Y and the Advancing Health Literacy to Enhance Equitable Community Responses to COVID-19 Initiative. This work enabled community council of the SDCOI to synthesize over 8,000 stories from 1,500 San Diegans into shared priorities for food, neighborhood, and health justice. This process illustrated how distinct forms of expertise could be integrated: Community Council Members contributed cultural and relational knowledge grounded in community trust, while researchers and clinicians brought methodological expertise to translate community priorities into actionable study designs. In addition, iterative checkpoints, shared leadership, and community review further underscored the importance of clarifying which forms of expertise should guide each stage of decision-making, ensuring that the project advanced inclusively and efficiently.

The Process evaluation focused on the clinical trial element of H4Y [58], directly influenced the co-development processes of co-leadership establishing in the American Heart Association-funded “Community Incubator Network” (KIN) project [59]. This project, which is a partnership housed within the San Diego County Childhood Obesity Initiative and includes both the YMCA of San Diego and UC San Diego, is co-led by both community Principal Investigators (PI), who are persons with high lived expertise and leadership history in community, and academic Principal Investigators, with high trained expertise. The team has been actively working collaboratively in ways that align with the dynamic decision-making process articulated here and has advanced a variety of approaches and strategies needed to support this. For example, within the KIN project, there was active work to re-imagine the notion of a “PI” to have a role that is appropriate for communities. This was done via taking a strengths-based and relationally driven orientation to mapping out the roles and responsibilities for each individual community PI. While neither H4Y nor KIN are DTx projects, both include active cultivation of teams that honor both lived and trained expertise in co-leadership of these broader efforts. These experiences were leveraged to guide the development of these worksheets to start to formalize what has been a more active and dynamic process in these areas, thus, enabling other groups to experiment with these types of approaches to co-leadership. These efforts reflect, in practice, what Ridgeway’s cultural schema theory of status predicts: status hierarchies can be disrupted when teams make relational processes explicit and consciously align esteem and authority with contextually relevant expertise. The lessons from H4Y and KIN informed how our Worksheet operationalizes this principle, turning what has been tacit relational work into an intentional, teachable process for dynamic expertise integration.

Taken together, these theoretical principles and real-world insights shaped our Worksheet into a practical tool for systematically recognizing diverse expertise, fostering inclusive and transparent decision-making, and promoting collective ownership across all phases of DTx development. In this way, the Worksheet is not only a synthesis of established frameworks but also a method refined through lived practice.

## A suggested approach

The Worksheet is designed to guide teams through essential activities that foster collaboration, alignment, and effective dynamic decision-making throughout the DTx development process. A detailed use case of this Worksheet is illustrated in S2 Table in S1 Table. The Worksheet includes a series of steps that help team members better understand each other’s roles, expertise, and perspectives, with a special focus on building mutual trust and respect among diverse team members that honors both lived and trained expertises each person has relevant to the targeted DTx. As described earlier, these activities are particularly important when working with populations that have been historically excluded in the development and adoption of DTx. The goal is to ensure that all voices are heard and valued, contributing to a more inclusive, effective DTx development process.

The Worksheet comprises of two sections: 1) DTx Team Building Steps and 2) Modules for DTx Team Activities. The first section is dedicated to team building, focusing on clarifying roles, aligning expectations, and fostering a shared understanding among team members—critical steps for establishing a solid foundation. The second section introduces five modules: a) Goal and Boundary, b) Milestone and Resources, c) Outcome, d) Benefit, and e) Product. These modules are not intended to be applied in a strictly linear sequence; instead, they are designed to guide decision-making dynamically, with different modules becoming more or less salient depending on the phase of the DTx lifecycle (see Framework described earlier) and the team’s evolving needs. This dynamic approach allows the modules to adapt to changes in team composition and ensures that the primary decision-makers and contributors for each activity are clearly identified, promoting effective and responsive decision-making.

## Fundamental components of the worksheet

### From Section 1. DTx team building steps

#### Assessing expertise.

A core activity in the DTx Team Building Worksheet is assessing the varying levels of expertise that each team member brings to the project. This includes both trained expertise (skills gained through formal education or professional experience) and lived expertise (knowledge gained from personal experience or community involvement). By acknowledging and incorporating both types of expertise, teams can make more informed and contextually relevant decisions. This step directly reflects the theoretical shift discussed in Part 2, from automatic to contextual status allocation, by emphasizing that influence should flow to those whose expertise, whether lived or trained, is most relevant to the decision at hand. In doing so, it operationalizes a key principle of our framework: making status dynamics explicit and aligning authority with contextual relevance. This activity also aligns with the CLT principle of empowerment and recognizing diverse knowledge systems. In DTx development, especially when engaging with diverse populations, integrating both professional and lived expertise ensures that solutions meet real-world needs, truly reflecting the values and priorities of the communities they are designed to serve. Furthermore, this process is consistent with the Inclusive Tech Design aspect articulated in the CCRD framework, which advocates for the systematic incorporation of community knowledge into design and development processes. This ensures that ethical considerations, cultural responsiveness, and community well-being are central to the design of digital health technologies [6]. Ultimately, the Worksheet promotes a participatory approach wherein team members contribute through both technical and lived experience, fostering inclusive, community-informed decision-making.

#### Defining roles and responsibilities.

Another essential activity in the worksheet is defining roles and responsibilities for each team member. This helps clarify who is responsible for what tasks, preventing overlap and misunderstandings, and ensuring more efficient collaboration that can remain adaptive throughout different phases of the DTx development and deployment. Conceptually, this reflects the theoretical shift outlined in in Part 2, from static hierarchy to dynamic influence. By making roles adaptive rather than fixed, teams can redistribute authority as different forms of expertise become salient during various stages of DTx development. This approach enables fluid collaboration, allowing decision-making power to move according to contextual expertise rather than formal credentials. This process is also in line with the CLT principle of a holistic and strength-based approach, which focuses on recognizing and leveraging complementary strengths within the team. By identifying each person’s unique skills and contributions, teams can create a cooperative, rather than competitive, environment. This principle reinforces the idea of fostering collective ownership, where each person’s strengths are valued, ultimately building a stronger, more cohesive team.

### From Section 2. Modules for DTx team activities

#### Identifying decision-making priorities for each module, activity, and phase.

**Fig 3:**
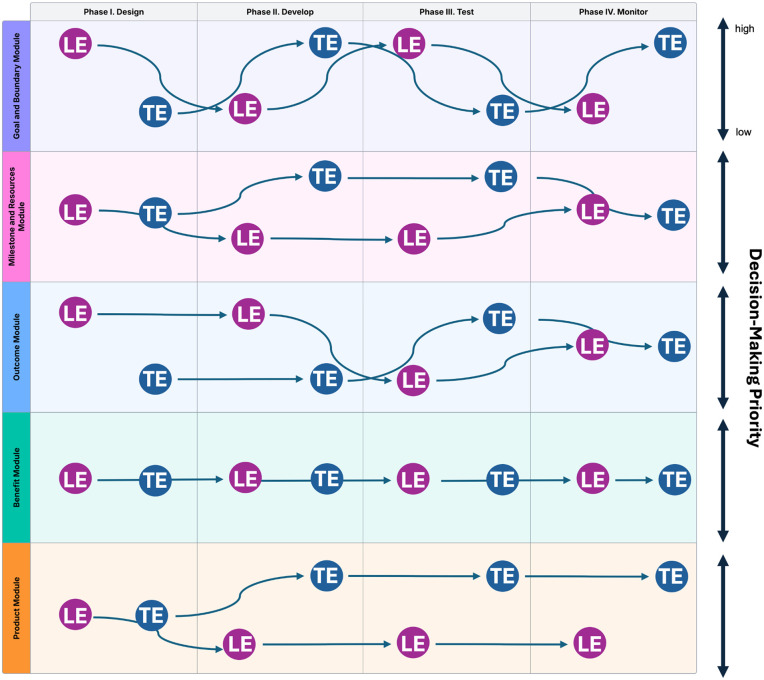
Example of dynamics in decision-making priority over time throughout the DTx lifecycle. The x-axis represents the four phases of the DTx lifecycle as described in the DTx RWE Framework (Phase I: Design, Phase II: Develop, Phase III: Test, and Phase IV: Monitor). The y-axis lists the modules from the Worksheet for DTx Team Activities, including the Product Module, Benefit Module, Outcome Module, Milestone and Resources Module, and Goal and Boundary Module. “LE” refers to an individual with lived expertise, while “TE” refers to an individual with trained expertise. The figure illustrates how, across different phases and modules, decision-making priority may shift toward the expertise most relevant for effectively advancing the project.

The second part of the worksheet outlines questions to determine which expertise(s) may guide decision-making for each module, activity, and phase (see Fig 3 as an example). This step operationalizes another core theoretical shift from Part 2, from implicit performance expectations to explicit expertise mapping. By clearly identifying which forms of expertise should guide specific decisions, teams counteract hidden biases tied to credentials or status and make the decision-making process more transparent and equitable. Prioritizing decision-making does not entail dominating discussions or excluding other perspectives. Rather, it involves identifying the individual(s) whose experience is most relevant to a particular discussion/decision to effectively advance the project. This method ensures that decisions are made transparently and inclusively, reflecting the diverse perspectives within the team throughout the entire DTx development and deployment process. Aligned closely with the CLT principles of codesign, being adaptive and responsive, and fostering sustainability, this approach emphasizes the need for collaborative solutions that are technically sound and widely acceptable and adaptable. Moreover, it contributes to the development of a sustainable ecosystem in which individuals and groups are supported by strong networks and infrastructures. This decision-making process also operationalizes the Community Partnership aspect of the CCRD framework by fostering ongoing, reciprocal engagement with community partners. Rather than approaching product development through an extractive lens—designing *for* communities—this component emphasizes shared ownership by designing *with* communities to ensure mutual benefit and long-term investment.

Together, these components operationalize the theoretical principles described earlier, contextual expertise recognition, dynamic influence, and explicit expertise mapping, by embedding them into everyday team practices. In doing so, the Worksheet translates abstract ideas about status and expertise into a structured, participatory process for building equitable and contextually responsive DTx teams.

## Applicability of the developed worksheet

### Who are the potential users of this worksheet?

This Worksheet can be used by anyone involved in any phase of the DTx Real-World Evidence (RWE) Framework—such as design, development, testing, and monitoring. This includes DTx providers (UI/UX designers, business managers, reimbursement experts), clinicians, patients, academics, and community stakeholders.

### When should this worksheet be applied?

This Worksheet is specifically crafted for use throughout all phases of the DTx lifecycle, including design, development, testing, and monitoring. The first section of the Worksheet is particularly useful at the start of a project, allowing groups to evaluate and ensure they have a robust team capable of managing the DTx design, development, testing, and monitoring processes. Initially, it’s essential to establish clear agreements on foundational project elements such as target users, health behaviors, and disease context. This section also helps define roles, responsibilities, and the diverse backgrounds and experiences of team members who are relevant to the project. This may ensure that the right people are in place with the necessary experience. Furthermore, this section can be revisited when adapting to changes in team structure or integrating new members, facilitating a dynamic approach to decision-making based on the expertise of latest team members.

The second section of the Worksheet is designed for continuous use throughout the DTx lifecycle. It guides teams through addressing the essential questions tied to each module, while matching which expertise has higher priority in decision-making responsibilities to answer those primary questions. This ensures detailed and relevant information is continually integrated into project planning across the DTx lifecycle. The flexibility of this section allows it to be adapted dynamically to meet the changing needs of the team and the project’s stage, ensuring that the modules can be applied as required by the evolving context.

### What are some potential benefits from using these tools?

While we have not formally tested this worksheet, as stated earlier, it was developed based on both strong prior theoretical and experiential foundations. From this, we do have a number of hypothesized benefits to its use, which others could examine and are in alignment with the success criteria flagged at the end of Part 2.

Firstly, we hypothesize that the tools reduce the likelihood of communication breakdowns and misunderstandings among team members and partners, which helps build trust within the team. Additionally, these tools promote clearer, more straightforward communication, which is essential for achieving project milestones and improving overall efficiency. By fostering a culture of trust and ownership, the tools encourage team members to value local knowledge and perspectives, enhancing the cultural responsiveness of the DTx product. This not only prevents harm and reduces bias but also builds empathy among the team, ensuring that decisions are made fairly and inclusively. Furthermore, the implementation of two-way feedback loops facilitates continuous engagement and adaptation, making the technology more aligned with the needs and values of diverse user groups. By streamlining communication and collaboration, these tools can save both time and resources, ultimately supporting the creation of a DTx product that is both relevant to its target users and sustainable in real-world settings.

### What additional factors should be considered when implementing the worksheet?

While the Worksheet provides the structural scaffolding for team alignment and decision-making, its success in practice also depends on attending to broader structural and implementation factors. Before the Worksheet can be applied, a critical factor to address is the process of team formation, with particular attention to recognizing and addressing historical imbalances in resources and objectives among potential actors. For example, the large technology companies often hold substantial economic and cultural influence, and relationships with smaller community groups have historically been uneven, which justifiably raises concerns about reciprocity. Nevertheless, when partners on both sides commit to transparency and shared values, including clear communication about potential competing interests, collaborations can generate new opportunities that might otherwise be inaccessible, including access to resources, visibility, and a mutual exchange of expertise.

In practice, a key part of team formation involves identifying and engaging community partners. Community actors are most effectively reached through existing local entities and infrastructures, such as community-based organizations, coalitions, and trusted cultural brokers (e.g., promotoras or community health workers). For example, both the H4Y project and subsequent KIN project, grew out of the San Diego County Childhood Obesity Initiative and the long-term work and commitment focused on centering community voice and priorities in that work [60].

To address scalability of this tool, teams may apply the Worksheet iteratively across different project scales, updating expertise mapping as membership evolves and using digital collaboration tools to maintain transparency. Because implementation requires resources beyond the Worksheet itself, it is important to plan dedicated budget lines for partner compensation and technical support. In the H4Y project, community partners were compensated through both direct incentives as tokens of appreciation (e.g., $25 gift cards and trainer certificates) and organizational subawards (e.g., funds to support community leaders’ salaries and coordination activities), illustrating one practical model for equitable resource allocation. It is also important to consider capacity-building approaches to support scalability and long-term sustainability. In the H4Y project, the coalition’s community partners were trained to map local health stories and then supported others in sharing their experiences. Using a train-the-trainer model and the multilingual, multimodal Streetwyze platform enhanced scalability, sustainability, and community ownership of the process.

Beyond compensation and capacity-building, teams also need strategies for addressing challenges that arise when different domains of expertise overlap. The Worksheet can serve as a practical tool for conflict resolution by clarifying which expertise should take the lead in specific decisions while recognizing the supporting roles of others. However, while this provides a useful strategy at the team level, broader structural barriers must also be acknowledged. To mitigate these barriers, projects should proactively attend to power dynamics, for example by adopting co-leadership or rotating facilitation models that ensure balanced participation across academic and community partners. One practical example is when community leaders are formally included as co-principal investigators on funded projects, positioning them not only as advisors but as equal decision-makers with shared authority and accountability, as was the case in the KIN project described earlier, and which was seeded with prior work [60]. Similarly, questions of intellectual property and data ownership should be addressed early through transparent agreements, preventing misunderstandings later in the process.

In addition, effective use of the Worksheet may require preparatory training and deliberate adaptation across phases of the DTx lifecycle. At project initiation, brief orientation sessions can help team members become familiar with the Worksheet’s decision-making framework, establish a shared vocabulary for valuing both lived and trained expertise, and practice strategies for navigating overlapping roles. As the project progresses, the Worksheet should be adapted to the specific needs of each phase: clarifying expertise and responsibilities during design, prioritizing technical and clinical decisions during development, incorporating community input and evaluation metrics during testing, and revisiting expertise mapping to reflect new partnerships or contexts during monitoring and sustainment. These practices ensure that the Worksheet functions not only as a structural guide but also as a living tool that evolves with the team and context.

## Discussion

Our work contributes to ongoing conversation in the DTx literature by addressing a gap between conceptual calls for inclusive, community-engaged approaches and the practical tools needed to implement them [5,61–63]. While prior studies and frameworks have highlighted the importance of representation, trust, and alignment with community values, many fall short of presenting actionable methods to systematically embedding these principles into the DTx development process. The Worksheet we propose directly addresses this gap by operationalizing concepts from Team Science [7,8], Community-Led Transformation [5], and Culturally and Community Responsive Design [6] into a structured, repeatable tool for practice.

In doing so, our findings help bridge a persistent divide in the DTx field, between innovation driven primarily by industry and research actors, and the lived realities and priorities of patients and communities. By explicitly incorporating both trained and lived expertise into dynamic decision-making across all phases of the DTx lifecycle, the Worksheet fosters conditions under which DTx products can be both technically rigorous and contextually responsive. Our work also offers actionable strategies for stakeholders seeking to enhance representation in DTx development and deployment. Specifically, the Worksheet can be applied at project initiation to ensure diverse expertise is present, roles and responsibilities are clearly defined, and decision-making priorities are transparent. It also supports practical implementation strategies such as leveraging existing community infrastructures for recruitment, establishing co-leadership models, and budgeting for equitable compensation of community partners. Together, these strategies provide a pathway toward more inclusive, sustainable, and trusted DTx.

## Limitations and proposed next steps

The DTx Team Building Worksheet, while promising, recognizes several key challenges that require actionable solutions. The main limitation is the need for empirical validation to establish the practical value of the Worksheet across varied DTx development contexts. To address these challenges, we recommend a two-pronged approach: First, we suggest organizing workshops and discussion forums to gather broad stakeholder feedback. This will be crucial for refining the Worksheet to better suit the dynamic needs of teams involved in DTx development. In this spirit, we want to invite other interested groups, including academia, industries, communities, healthcare clinics, regulatory policy makers, insurance companies, and others, to work with us on effective partnership strategies that center community voices. Second, pilot testing the Worksheet in real-world projects should be pursued concurrently. This pilot testing will not only provide empirical validation but also allow for the immediate application of feedback, enhancing the Worksheet’s effectiveness and ensuring it meets diverse organizational needs. Securing funding through initiatives like ComPASS Community Engagement will support these activities, facilitating necessary training, collaborations, and adaptations of the Worksheet. This integrated approach of feedback, testing, and adaptation will ensure the Worksheet is both effective and applicable in diverse settings, ultimately supporting robust and sustainable DTx development.

## Conclusion

Digital therapeutics hold tremendous promise for addressing unmet healthcare needs across diverse populations, yet current approaches to DTx development systematically reproduce rather than reduce health inequities. This paper has identified a fundamental problem, articulated a theoretical framework for addressing it, and offered a practical tool for operationalizing that framework in real-world DTx development contexts.

We expanded the notion of the digital divide beyond simple access issues to encompass four interconnected domains: the Digital Knowledge Divide in understanding how to produce community-relevant interventions, the Digital Evidence Generation Divide in whose data informs algorithm development and validation, the Digital Production Divide in who shapes design and development decisions, and the Digital Adoption Divide in differential uptake and sustained engagement. These divides reveal that inequities in DTx stem less from technical limitations than from the systematic exclusion of lived expertise from the design and decision-making process.

To address, we drew on Ridgeway’s cultural schema theory of status to explain how conventional team hierarchies in DTx development allocate status and decision-making authority through automatic cultural defaults, credentials, professional roles, demographic characteristics, rather than through contextual assessment of who possesses the most relevant expertise for specific decisions. This represents a fundamental misalignment between status allocation and actual contribution to the shared goal of developing equitable, effective interventions. Our theoretical framework encourages teams to explicitly examine and renegotiate what counts as legitimate expertise in context, creating conditions where influence flows toward those with the most relevant understanding for each decision.

We operationalized this theoretical approach through the DTx Team Building Worksheet, a practical tool that integrates team science, Community-Led Transformation, and Culturally and Community Responsive Design principles. The Worksheet guides teams to dynamically assess expertise, adapt decision-making roles across project phases, and make implicit hierarchies explicit, enabling fluid rather than fixed collaboration structures. This is not merely an idealistic aspiration; our collaborative work has demonstrated that such dynamic processes are achievable when teams commit to making implicit hierarchies explicit and developing shared understanding of distributed expertise.

Together, these three contributions, the expanded conceptualization of digital divides, the status-based theoretical framework for dynamic expertise integration, and the practical Worksheet tool, offer a coherent pathway for making DTx development more culturally and contextually responsive. Yet we recognize that this work is in its pilot stage and requires broader validation, refinement, and adaptation. We invite stakeholders across academia, industry, community organizations, healthcare systems, regulatory bodies, and insurance companies to engage with these tools, test them in diverse contexts, and contribute to their evolution. The ultimate measure of success will not be whether teams adopt this specific Worksheet, but whether the broader field develops capacity for the kind of reflexive, inclusive, dynamically responsive partnership practices that can produce DTx truly designed for and with the communities they aim to serve. In this way, addressing the digital divides in DTx becomes not only a matter of fairness, but also an opportunity to model more accessible approaches to technology development that could extend beyond digital therapeutics to transform how we create interventions across the health technology landscape.

## Supporting information

S1 Text DTx team building worksheet contents. (DOCX)

S1 Table Supplementary tables. (XLSX)
