# Supplementary material for: Bridging the divide in digital therapeutics (DTx): Partnership strategies for broader representation across DTx development and deployment
Source: PLOS Digit Health. 2026 Feb 20;5(2):e0001241. doi: 10.1371/journal.pdig.0001241 (PMC12922973; doi:10.1371/journal.pdig.0001241)
Supplement: S1 Text — (DOCX) [file pdig.0001241.s001.docx]

**DTx Team Building Worksheet Contents**

**Part 1. DTx team building steps:**

1. Meeting each other
   1. Understanding each group of the DTx team
   2. Understanding each individual of the DTx team
2. Learning about the project
3. Planning how we’ll do the project

**Part 2. Modules for DTx team activities:**

| **Module** | **What do we do** | **Primary questions to think about** | **Decision Priority Status** |
| --- | --- | --- | --- |
| Goal and boundary module | Refine the main goals & boundaries of the project | - What is our goal in each activity in every phase of the DTx RWE Framework? | Lived expertise |
|  |  | - What is our boundary in each activity and phase of the DTx RWE Framework? |  |
| Milestone and resources module | Check the milestones & resources of the project | - What steps must we take to achieve our goals and what is the initial timeline for each activity and phase of the DTx RWE Framework? | Both lived and trained expertise |
|  |  | - What things do we need to meet our goals in each activity and phase of the DTx RWE Framework? |  |
| Outcome module | Identify the expected outcomes of the project | - What’s the best thing (e.g., meaningful  benchmark) we want from our DTx via each activity and phase of the DTx RWE Framework? | Lived expertise |
|  |  | - What are other good things we hope to see from each activity and phase of the DTx RWE Framework? |  |
| Benefit module | Talk about what the personal benefit might be like | - What personal gains do you see coming from your involvement in each activity and phase of the DTx RWE Framework? | Both lived and trained expertise |
|  |  | - Are the expectations everyone has from each activity and phase of the DTx RWE Framework reasonable in your view? |  |
| Product module | Discuss which features or components we will add to our product | - What parts or features might we add to our product from each activity and phase of the DTx RWE Framework? | Trained expertise |
|  |  | - How can we check if our product from each activity and phase of the DTx RWE Framework works well and really helps people? |  |

**DTx Team Building Steps**

1. **Meeting each other**

**1-a. Understanding each group of the DTx team**

- **Purpose:** To cultivate understanding of each group by finding out who are they, what they care about (their values), what they want to achieve (their goals), what special thing they can do (their assets)

**What you need:** A list of potential groups who are interested in being a DTx team

**What you’ll do:** Learn about each group who are potentially going to be a part of DTx team

**What you’ll get:** Which group will get involved to form a DTx team

Please briefly introduce your group to others by answering the following questions:

- **Group 1**
  - Name:
  - What does your group care about most?
  - What does your group want to achieve?
  - What special things can your group bring to this project?
- **Group 2**
  - Name:
  - What does your group care about most?
  - What does your group want to achieve?
  - What special things can your group bring to this project?
- **Group 3**
  - Name:
  - What does your group care about most?
  - What does your group want to achieve?
  - What special things can your group bring to this project?
- **Group 4**
  - Name:
  - What does your group care about most?
  - What does your group want to achieve?
  - What special things can your group bring to this project?
- **Group 5**
  - Name:
  - What does your group care about most?
  - What does your group want to achieve?
  - What special things can your group bring to this project?

If there are more groups, please add them accordingly.

**1-b. Understanding each individual of the DTx team**

- **Purpose:** To learn more about everyone on our team by finding out what they know and what they have experienced that can help our project.

**What you need:** A list of all the people from each group who will be part of the DTx team

**What you’ll do:** Learn about each person who might join the DTx team

**What you’ll get:** Which individual will be part of the DTx team

Please complete the form below for each person.

***About your relevant trained expertise:***

**1) What specific skills from your formal training could be helpful for this project?**

- 1. Skill # 1
  2. Skill # 2
  3. Skill # 3

**2) How would you rate your expertise in the trained skills you’ve mentioned above?**

**Response option:**

**0 = No experience,**

**1 = Beginner,**

**2 = Intermediate,**

**3 = Advanced,**

**4 = Expert**

- 1. Skill # 1 – your level of expertise:
  2. Skill # 2 – your level of expertise:
  3. Skill # 3 – your level of expertise:

**3) What led you to rate your trained expertise in trained skill that way?**

***About your relevant lived expertise:***

**1) What personal lived experiences do you have that are important for developing, testing, and maintaining the DTx?**

- 1. Lived expertise #1
  2. Lived expertise #2
  3. Lived expertise #3

**2) How would you rate your level of lived expertise based on your lived experiences?**

**Response option:**

**0 = No experience,**

**1 = Having/Had a close friend/family member/colleague experiencing the target identity/phenomena,**

**2 = Living/Lived with a person with the target identity/phenomena,**

**3 = Experiencing/experienced the target identity/phenomena,**

**4 = Engaged in organizing and engaging with others with the target identity/phenomena; feel confident in representing diverse perspectives among those who experience the targeted identity/phenomena**

- 1. Lived expertise #1 – your level of expertise:
  2. Lived expertise #2 – your level of expertise:
  3. Lived expertise #3 – your level of expertise:

**3) What led you to rate your lived expertise in lived experiences that way?**

1. **Learning about the project**

**Understanding each major task of the DTx team**

- **Purpose:** to help everyone understand the main tasks of the project and who will lead each one

**What you need:** Learn about the three major tasks in making and using the DTx

**What you’ll do:** Find out who will lead each of these major tasks

**What you’ll get:** A list of the people who will lead each major task

To start, let’s learn about the three major tasks in making and using the DTx.

**The list of 3 major tasks:**

- ***DTx Builders: These are the people who make and manage the DTx.***
  1. Think of them like the developers of a video game, but for health programs. They can be people who work at places such as DTx company, medical centers, or public health departments with proven software development and management capabilities
  2. Relevant expertise: trained expertise and lived expertise
- ***DTx Users: These are the people who are going to use the DTx.***
  1. They help by giving information about how the DTx works when real people use it in the real world. These can be individual patients, local clinic workers, doctors or nurses in clinics, or people who work at places where they help lots of others stay healthy and collect this important real-world information.
  2. Relevant expertise: lived expertise
- ***DTx Checkers:*** ***These are the people who check to make sure the DTx works right as intended.***
  1. They use their knowledge from school and work to make sure the DTx is helpful and safe. They could be people who work at universities, research centers, or organizations that know a lot about health research studies.
  2. Relevant expertise: trained expertise

Then, let’s find out who will lead each major task in making and using the DTx.

Who are the people leading the task of

- **DTx Builders:**
- **DTx Users:**
- **DTx Checkers:**

1. **Planning how we will do the project**

**Establishing overall rule of the DTx team for this project**

- **Purpose:** to define how team members should communicate to keep the team responsive and engaged

**What you need:** Be ready to share any existing rules (things we can't do) that everyone needs to know

**What you’ll do:** Discuss which communication rules will help our team activities the most

**What you’ll get:** A set of communication rules that everyone agrees on and that helps everyone during our team activities

**3-a. Communication protocols:**

- Communication platform:
- Primary contact points for each task:
  - DTx implementor:
  - User-serving organizations:
  - DTx evaluator:
- Communication schedule:
  - Set regular meeting times (that all team members across different time zones):
  - The frequency of all-team meetings (daily, weekly, bi-weekly):

**3-b. Documentation and record keeping:**

- How will project documents be shared?
- Where will project documents be stored?

**Modules for DTx team activities**

**[Overview of the DTx team activities]**

| **Module** | **What do we do** | **Primary questions to think about** | **Decision Priority Status** |
| --- | --- | --- | --- |
| Goal and boundary module | Refine the main goals & boundaries of the project | - What is our goal in each activity in every phase of the DTx RWE Framework? | Lived expertise |
|  |  | - What is our boundary in each activity and phase of the DTx RWE Framework? |  |
| Milestone and resources module | Check the milestones & resources of the project | - What steps must we take to achieve our goals and what is the initial timeline for each activity and phase of the DTx RWE Framework? | Both lived and trained expertise |
|  |  | - What things do we need to meet our goals in each activity and phase of the DTx RWE Framework? |  |
| Outcome module | Identify the expected outcomes of the project | - What’s the best thing (e.g., meaningful  benchmark) we want from our DTx via each activity and phase of the DTx RWE Framework? | Lived expertise |
|  |  | - What are other good things we hope to see from each activity and phase of the DTx RWE Framework? |  |
| Benefit module | Talk about what the personal benefit might be like | - What personal gains do you see coming from your involvement in each activity and phase of the DTx RWE Framework? | Both lived and trained expertise |
|  |  | - Are the expectations everyone has from each activity and phase of the DTx RWE Framework reasonable in your view? |  |
| Product module | Discuss which features or components we will add to our product | - What parts or features might we add to our product from each activity and phase of the DTx RWE Framework? | Trained expertise |
|  |  | - How can we check if our product from in each activity and phase of the DTx RWE Framework works well and really helps people? |  |

**Goal and boundary module**

- **What do we do:** Refine the main goals & boundaries of the project
- **Primary questions to think about in this module:**
  - What is our goal in each activity in every phase of the DTx RWE Framework?
  - What is our boundary in each activity and phase of the DTx RWE Framework?

Before we start talking about this module, let’s first decide how we will make decisions for this part of our project.

***Decision-making process for this module: Deciding Who Makes Decisions***

1) Please answer the following questions to help us figure out who should make decisions in this module of our project:

• Who knows the most about getting the right answers to our main questions?

• Who understands best what will happen because of the decisions we make?

• Who will be affected by these decisions, and have we asked them what they think?

2) Which person got picked the most in our questions above?

3) What types of expertise does this person have? Is it from training (trained expertise) or from what they’ve lived through (lived expertise)?

4) Does their expertise match what we said about “who decides” in our guidelines?

**Milestones and resources module**

- **What do we do:** Set the milestones & resources of the project
- **Primary questions to think about in this module:**
  - What steps must we take to achieve our goals and what is the initial timeline for each activity and phase of the DTx RWE Framework?
  - What things do we need to meet our goals in each activity and phase of the DTx RWE Framework?

Before we start talking about this module, let’s first decide how we will make decisions for this part of our project.

***Decision-making process for this module: Deciding Who Makes Decisions***

1) Please answer the following questions to help us figure out who should make decisions in this module of our project:

• Who knows the most about getting the right answers to our main questions?

• Who understands best what will happen because of the decisions we make?

• Who will be affected by these decisions, and have we asked them what they think?

2) Which person got picked the most in our questions above?

3) What types of expertise does this person have? Is it from training (trained expertise) or from what they’ve lived through (lived expertise)?

4) Does their expertise match what we said about “who decides” in our guidelines?

**Outcome module**

- **What do we do:** Identify the expected outcomes of the project
- **Primary questions to think about in this module:**
  - What’s the best thing (e.g., meaningful benchmark) we want from our DTx via each activity and phase of the DTx RWE Framework?
  - What are other good things we hope to see from each activity and phase of the DTx RWE Framework?

Before we start talking about this module, let’s first decide how we will make decisions for this part of our project.

***Decision-making process for this module: Deciding Who Makes Decisions***

1) Please answer the following questions to help us figure out who should make decisions in this module of our project:

• Who knows the most about getting the right answers to our main questions?

• Who understands best what will happen because of the decisions we make?

• Who will be affected by these decisions, and have we asked them what they think?

2) Which person got picked the most in our questions above?

3) What types of expertise does this person have? Is it from training (trained expertise) or from what they’ve lived through (lived expertise)?

4) Does their expertise match what we said about “who decides” in our guidelines?

**Benefit module**

- **What do we do:** Talk about what the personal benefit might be like
- **Primary questions to think about in this module:**
  - What personal gains do you see coming from your involvement in each activity and phase of the DTx RWE Framework?
  - Are the expectations everyone has from each activity and phase of the DTx RWE Framework reasonable in your view?

Before we start talking about this module, let’s first decide how we will make decisions for this part of our project.

***Decision-making process for this module: Deciding Who Makes Decisions***

1) Please answer the following questions to help us figure out who should make decisions in this module of our project:

• Who knows the most about getting the right answers to our main questions?

• Who understands best what will happen because of the decisions we make?

• Who will be affected by these decisions, and have we asked them what they think?

2) Which person got picked the most in our questions above?

3) What types of expertise does this person have? Is it from training (trained expertise) or from what they’ve lived through (lived expertise)?

4) Does their expertise match what we said about “who decides” in our guidelines?

**Product module**

- **What do we do:** Discuss which features or components we will add to our product
- **Primary questions to think about in this module:**
  - What parts or features might we add to our product from each activity and phase of the DTx RWE Framework?
  - How can we check if our product from in each activity and phase of the DTx RWE Framework works well and really helps people?

Before we start talking about this module, let’s first decide how we will make decisions for this part of our project.

***Decision-making process for this module: Deciding Who Makes Decisions***

1) Please answer the following questions to help us figure out who should make decisions in this module of our project:

• Who knows the most about getting the right answers to our main questions?

• Who understands best what will happen because of the decisions we make?

• Who will be affected by these decisions, and have we asked them what they think?

2) Which person got picked the most in our questions above?

3) What types of expertise does this person have? Is it from training (trained expertise) or from what they’ve lived through (lived expertise)?

4) Does their expertise match what we said about “who decides” in our guidelines?
